# Human Mesenchymal Stem Cell (hMSC) Donor Potency Selection for the “First in Cystic Fibrosis” Phase I Clinical Trial (CEASE-CF)

**DOI:** 10.3390/ph16020220

**Published:** 2023-02-01

**Authors:** Tracey L. Bonfield, Morgan T. Sutton, David R. Fletcher, Jane Reese-Koc, Erica A. Roesch, Hillard M. Lazarus, James F. Chmiel, Arnold I. Caplan

**Affiliations:** 1 Department of Genetics and Genome Sciences, National Center Regenerative Medicine and Pediatrics, Case Western Reserve University School of Medicine, 10900 Euclid Avenue, BRB 822, Cleveland, OH 444106, USA; 2 National Center for Regenerative Medicine, Case Western Reserve University School of Medicine, Cleveland, OH 444106, USA; 3 Department of Pediatric Pulmonary, Rainbow Babies and Children’s Hospital, Cleveland, OH 44106, USA; 4 Saint Jude Children’s Research Hospital, Graduate School of Biomedical Sciences, Memphis, TN 38105, USA; 5 University Hospitals Seidman Cancer Center, Cleveland, OH 44106, USA; 6 Department of Pediatrics, Riley Hospital for Children at IU Health, Indiana University School of Medicine, Indianapolis, IN 46202, USA; 7 Skeletal Research Center, Department of Biology, Case Western Reserve University, Cleveland, OH 44106, USA

**Keywords:** human mesenchymal stem cells, anti-inflammatory potency, anti-microbial potency, cystic fibrosis, clinical trial development

## Abstract

Human Mesenchymal Stem Cell (hMSC) immunotherapy has been shown to provide both anti-inflammatory and anti-microbial effectiveness in a variety of diseases. The clinical potency of hMSCs is based upon an initial direct hMSC effect on the pro-inflammatory and anti-microbial pathophysiology as well as sustained potency through orchestrating the host immunity to optimize the resolution of infection and tissue damage. Cystic fibrosis (CF) patients suffer from a lung disease characterized by excessive inflammation and chronic infection as well as a variety of other systemic anomalies associated with the consequences of abnormal cystic fibrosis transmembrane conductance regulator (CFTR) function. The application of hMSC immunotherapy to the CF clinical armamentarium is important even in the era of modulators when patients with an established disease still need anti-inflammatory and anti-microbial therapies. Additionally, people with CF mutations not addressed by current modulator resources need anti-inflammation and anti-infection management. Furthermore, hMSCs possess dynamic therapeutic properties, but the potency of their products is highly variable with respect to their anti-inflammatory and anti-microbial effects. Due to the variability of hMSC products, we utilized standardized in vitro and in vivo models to select hMSC donor preparations with the greatest potential for clinical efficacy. The models that were used recapitulate many of the pathophysiologic outcomes associated with CF. We applied this strategy in pursuit of identifying the optimal donor to utilize for the “First in CF” Phase I clinical trial of hMSCs as an immunotherapy and anti-microbial therapy for people with cystic fibrosis. The hMSCs screened in this study demonstrated significant diversity in antimicrobial and anti-inflammatory function using models which mimic some aspects of CF infection and inflammation. However, the variability in activity between in vitro potency and in vivo effectiveness continues to be refined. Future studies require and in-depth pursuit of hMSC molecular signatures that ultimately predict the capacity of hMSCs to function in the clinical setting.

## 1. Introduction

Cystic fibrosis (CF), the most common life-limiting genetic disease in Caucasians, is characterized by dysfunction of the cystic fibrosis transmembrane conductance regulator (CFTR) protein [1,2]. Mutations in the *Cftr* gene result in a wide range of phenotypes and severity in people with CF, but in most cases, patients suffer from severe pathophysiologic consequences and experience premature mortality. The advent of small molecule correctors and potentiators, known as modulators, have changed CF survival, and disabling morbidity [3,4]. However, the ability of modulators to reverse permanent damage in established lung disease is limited. The hMSC may provide a supportive therapeutic to manage both infection and inflammation in the context of permanent damage and on-going inflammation even with modulator therapy. Further, not all mutations in CF respond to small molecule therapy, and some patients do not tolerate the drugs well. The current initiatives are to develop new therapies for these CF anomalies while at the same time pursuing gene corrective technologies. A major issue in therapeutic management in CF is treating inflammation in the presence of active infection without exacerbating the infection [5,6].

Human mesenchymal stem cells (hMSCs) are “medicinal signaling cells” which secrete bioactive molecules capable of directly suppressing both inflammation and infection [7,8]. The hMSCs and their secreted products directly impact the tissue environment as well as re-direct immune cell phenotype, which can orchestrate tissue regeneration and repair damage [7,9]. Further, hMSCs have been effectively utilized in more than 1000 clinical trials internationally, without an associated significant adverse profile [10,11]. The therapeutic impact of hMSCs in many of these trials is due to their anti-inflammatory and anti-microbial effects. Although clinical benefit often may be impressive, there are a significant number of subjects in these trials who exhibit a sub-optimal response to hMSC treatment [12]. The variability in the hMSC treatment outcomes is due to not only the variability of hMSC products but also the disease in which the treatment is utilized [13,14,15]. In our studies, we have documented that not all hMSCs “are created equal” and that each donor hMSC has a unique functional profile which can be harnessed for potency and efficacy [13,16]. In preparation for a phase I clinical trial of hMSCs in subjects with CF, we systematically profiled eight different bone-marrow-derived hMSC preparations from healthy volunteers using good manufacturing practices (GMP) to identify which of the hMSCs would have the highest potential to provide a clinical benefit in our patient population.

The potency assays for our pre-clinical studies were focused on the capacity to manage infection and inflammation associated with CF [13,16]. In these studies, we have established the correlation between monitoring the in vitro hMSC secretome functional activity as a low-cost potency test for more expensive in vivo validations. The models included in vitro human and murine cells to provide systematic translation from pre-clinical modeling. The two hMSC preparations with the best in vitro potency were evaluated for their ability to attenuate infection and inflammation in the whole organism using a murine model of *Pseudomonas aeruginosa* (*P. aeruginosa*) infection. These models have been established as useful tools for evaluating therapeutics aimed at attenuating infection and inflammation [17,18,19]. Additionally, the hMSCs were cultured and managed in our own GMP facility and utilized the identical medium and conditions that would be recapitulated in the CF clinical trial. The data obtained from these hMSC in vitro and murine in vivo potency assays were used to select the hMSC donor preparation with the greatest potential to provide both anti-inflammatory and anti-microbial potency for the CEASE-CF Clinical Trial (NCT02866721) recently accepted for publication [20].

## 2. Results

### 2.1. hMSC Anti-Inflammatory Activity on BMDM Target Cells

**Figure 1:**
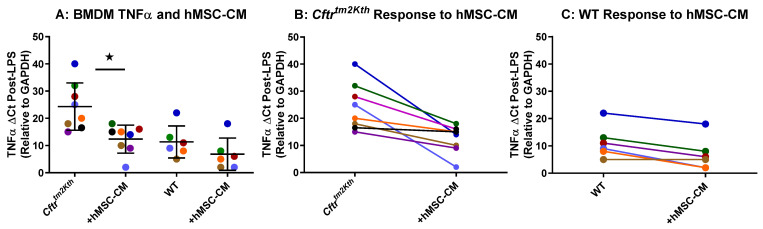
hMSC Anti-Inflammatory Potency Screen on Murine BMDM. Each of the pre-GMP donor preparations (shown by a different color) were cultured in the absence of antibiotics for 72 h. The supernatants were screened for the ability to suppress the production of TNFα from either murine WT or *Cftr^tm2Kth^* BMM stimulated with LPS (10 µg/mL, *n* = 8) (**A**). Evaluating each hMSC-CM individually, the *Cftr*-deficient (**B**, *n* = 8) and WT (**C**, *n* = 6) were analyzed. Consistent with previous studies, the hMSCs significantly decreased TNFα gene expression by the BMDM stimulated with LPS (star designates *p* < 0.05) with each hMSC-conditioned medium having a unique anti-inflammatory potency.

BMDM were obtained from the femurs of CF and WT mice and allowed to mature for 7 days. At 7 days, BMDM were cultured for 24 h with and without LPS and/or supernatant from one of each of the eight different hMSC-conditioned media (hMSC-CM). Both *Cftr*-deficient (*Cftr^tm2kth^*) and WT target cells were utilized to assure the capacity of the hMSC to be efficient in the unique cell settings. Each hMSC-CM was tested in duplicate on two different sets of BMDM and followed for BMDM TNFα response to LPS in the presence and absence of the hMSC-conditioned medium (Figure 1A). The LPS-induced significant levels of both TNFα by the Cftr-deficient bone marrow (Figure 1B, *p* < 0.05) and wild type bone marrow (Figure 1C) target cells were suppressed by the hMSCs.

### 2.2. hMSC Anti-Inflammatory Activity on Airway Epithelial Cells

**Figure 2:**
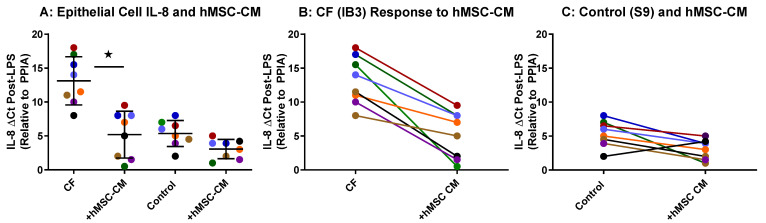
hMSC Anti-Inflammatory Potency Screen on Transformed Human Airway Epithelial Cells. Each of the pre-GMP donor preparations (shown by a different color) were cultured in the absence of antibiotics for 72 h. The supernatants were screened for the ability to suppress the production of IL-8 from either CF (IB3) or non-CF (S9) airway epithelial cell lines stimulated with LPS (10 µg/mL) (**A**). Evaluating each hMSC-CM individually, the CFTR-deficient cell line (**B**, IB3: *n* = 8) and control (**C**, S9: *n* = 8) were analyzed. Consistent with previous studies, the hMSCs significantly decreased IL-8 gene expression (star designates *p* < 0.05), with each hMSC condition medium having a unique anti-inflammatory potency.

The hMSC donor anti-inflammatory potency was quantified using transformed CF airway epithelial cells as previously described [16,21]. Both CFTR-deficient (CF) and control target cells were utilized to assure the capacity of the hMSC to be efficient in the unique CFTR-induced inflammatory response. Cell lines were utilized over primary airway epithelial cells since the cells and their inflammatory response was usedfor monitoring the hMSCs’ anti-inflammatory potency on the epithelial inflammatory response itself and not on the intrinsic defects of CFTR defects. However, primary cells are essential for defining the mechanisms associated with hMSC effects and are an on-going initiative currently. CFTR-deficient epithelial cells produce excessive IL-8 in response to LPS compared to the CFTR-corrected control cell line which is used to monitor the capacity of the hMSC to suppress IL-8 production. Utilizing these models of epithelial inflammation, the pre-GMP hMSCs were analyzed for functional anti-inflammatory activity (Figure 2). Each of the donor hMSC-conditioned media (shown by a different color) demonstrated variable anti-inflammatory effectiveness in both CFTR-deficient and control models of inflammation (Figure 2A). Each of the eight donor hMSC preparations decreased the LPS-induced epithelial cell transcription of IL-8 in the CF cell line (IB3) (Figure 2B, *p* < 0.05, *n* = 8) and the control cell line (S9, *n* = 8). As with the BMDM mouse cell line studies, each of the hMSC-derived conditioned medium sources demonstrated a different anti-IL-8 epithelial cell cytokine effect.

### 2.3. Anti-Microbial Potency of hMSCs against P. aeruginosa

**Figure 3:**
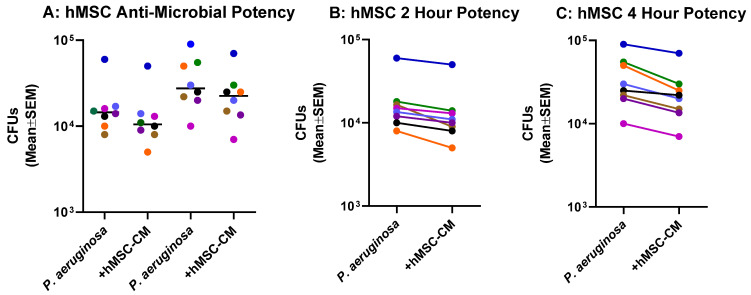
hMSC Anti-*P. aeruginosa* Potency Screen. Each of the hMSC-conditioned medium preparations generated without antibiotics were analyzed for antimicrobial potency against *Pseudomonas aeruginosa*. The hMSC-conditioned medium from each of the donors (shown by a different color) had antimicrobial potency (**A**). When each hMSC-conditioned medium preparations were analyzed individually for antimicrobial potency measuring CFUs at 2 h (**B**, *n* = 8) and 4 h (**C**, *n* = 8).

**Figure 4:**
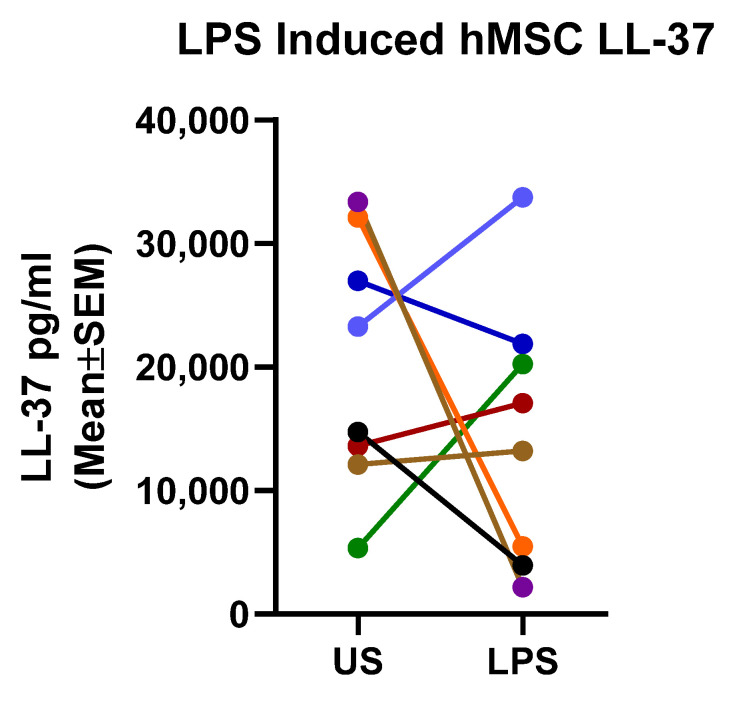
hMSC LL-37 Production. Each of the hMSCs were screened for the ability to secrete the anti-microbial peptide LL-37 in the presence and absence of a direct stimulation with LPS (10 µg/mL, *n* = 8). All eight pre-GMP hMSC donors secreted LL-37 (20,225 ± 9499 pg/mL, mean ± SD).

Each of the donor hMSC preparations were screened for the ability to reduce *P. aeruginosa* colony forming units (CFUs) as a measure of anti-microbial activity (Figure 3). Each of the hMSC preparations demonstrated some anti-*P. aeruginosa* effect at both 2 h (Figure 3A) and 4 h (Figure 3B), demonstrating the requirement for following hMSCs for sustainability of function. 

### 2.4. Mechanistic Fingerprinting of the Pre-GMP Donor Preparations

To begin to define the molecular signature of the ideal hMSC with optimal anti-inflammatory and antimicrobial activity, each of the hMSCs were screened for LL-37 (Figure 4). Not surprisingly, the pre-GMP hMSCs produced significantly variable concentrations of LL-37 at baseline and post-response to LPS (Figure 4). Analysis of the LPS-induced LL-37 concentration versus the antimicrobial potency demonstrated significance (*n* = 8, *p* = 0.02, r = 0.081) using nonparametric ANOVA analysis, but only at 2 h. LPS-induced LL-37 concentration versus the antimicrobial potency at 4 h had an r = 0.67, *p* = 0.07 (*n* = 8).

### 2.5. In Vivo Testing of Each of the Donor hMSCs

**Figure 5:**
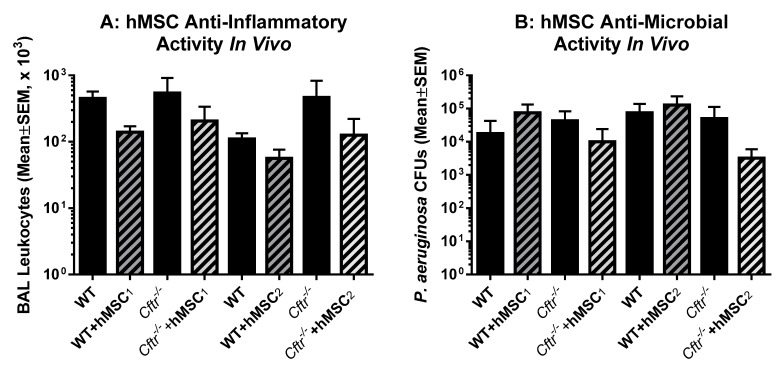
hMSCs in vivo Potency. Two of the pre-GMP hMSCs (hMSC_1_ and hMSC_2_) were selected for their anti-microbial potency and LL-37 production and studied in the murine model of CF *P. aeruginosa* lung infection using *n* = 5–6 mice in each group. Both hMSC preparations had anti-inflammatory (**A**) and antimicrobial (**B**) activity in vivo, but neither reached statistical significance. In comparing the “F” variance, hMSC_2_ has significant *p* values for both the anti-microbial and anti-inflammatory outcome measures. (**A**): *p* ≤ 0.05, total numbers of bronchoalveolar lavage leukocytes; (**B**): *p* ≤ 0.05, total number of lung *P. aeruginosa* CFUs.

Profiling hMSCs in vitro is a useful and cost-effective way of monitoring the potency of different hMSC preparations, providing insight into the phenotype and functional signatures. To ascertain the in vivo functionality of the pre-GMP hMSCs, we utilized the same in vivo modeling system used to generate the IND supporting pre-clinical data (Figure 5). To determine the in vivo efficacy of our designated “ideal” hMSC donor, two of the pre-GMP preparations (blue and red) were screened for the ability to improve the outcomes in the in vivo murine *P. aeruginosa* agarose bead model. CF and WT mice were inoculated with pseudomonas agarose beads and followed for 10 days at which time the mice were euthanized and evaluated for lung inflammatory markers, including bronchoalveolar lavage fluid white cell counts (Figure 5A) and total lung *P. aeruginosa* CFUs (Figure 5B).

The two different hMSC preparations demonstrated trends towards decreasing both white cell counts and total lung CFUs but did not reach statistical significance. This effect may be due to the variability in the lung infection modeling system or, importantly, the selection criteria hypothesized to be essential in defining the underlying in vivo potency. An evaluation of the groups with and without hMSC treatment for F variance differences demonstrated significance for hMSC_2_ with respect to both anti-inflammatory and anti-microbial potency (*p* ≤ 0.05).

## 3. Discussion

In previous pre-clinical studies, we evaluated the potential of hMSCs to treat the infection and inflammation that is typically associated with CF lung disease [16,21,22]. In these studies, we established the significant variability in effectiveness of hMSCs from different donors. In preparation for the “First in CF” clinical trial, eight different hMSC preparations were screened for potency using in vitro and in vivo models of CF lung infection-induced inflammation. In CF, the conundrum is reducing the host inflammatory response in the presence of an active bacterial infection. The therapeutics aimed at just the inflammatory piece of the CF pathophysiology have been problematic in providing a therapeutic benefit due to concurrent issues with pulmonary infection [1,5,23]. Given the unique pathophysiology of CF, hMSCs were screened for anti-microbial activity against *P. aeruginosa*, anti-inflammatory activity and production of the antimicrobial LL-37. The hMSC preparation which demonstrated the optimal anti-inflammatory and antimicrobial activities with sustained LL-37 production in response to LPS stimulation was selected for the phase I clinical trial. Of note, the preparation that was ultimately chosen for the clinical trial was from a donor who was CMV negative and met all other FDA screening requirements.

hMSC are a relatively novel and exciting therapeutic option for the treatment of chronic inflammation and infection in CF given their unique capacity to respond and actively contribute to the host environment [24]. The differences between hMSCs and other therapeutics lies in the hMSC paracrine capabilities and responsiveness to their environment which contribute to their clinical effectiveness [25]. Many subjects enrolled in hMSC clinical trials demonstrate a clinical response, but there is also a significant non-responder population that does not realize the same benefits from the therapy [12,26,27]. The effectiveness of hMSCs is dictated by the potency of the hMSCs themselves, the disease under treatment, and the severity of the disease that is being impacted. The validation of “bench-to-bedside” modeling systems is essential to identifying the most optimal and reproducible assays of clinical potency. 

Because the treatment of inflammatory diseases with hMSCs has been met with variability in terms of clinically significant outcomes [10,28,29], our studies were aimed at optimizing the hMSC donor selection to enhance the potential for providing a clinical benefit in the context of CF lung infection and inflammation. Defining the pre-clinical potency and efficacy of hMSCs using in vitro and in vivo models such as the murine model of CF lung-infection-induced inflammation provides insight into the potential for therapeutic efficacy but still does not guarantee the outcome because of the variability of the disease and patient characteristics. Even within our selection process to determine which hMSC donor preparation to test in the murine in vivo model, the criteria would be interpreted in more than one way, ultimately resulting in a different selection process. The implication here is that consideration must be given to the environment in which the hMSC product will be delivered and how it might differ between in the in vitro model and the complexity of the in vivo environment. Furthermore, other types of in vivo models such as the CF pig or ferret may provide an additional pre-clinical predictor given that these models better mimic the intrinsic CF pathophysiology associated with deficient CFTR [30,31]. The introduction of patient ex vivo tissue samples would provide additional strength for translating into clinical trials (13,14). Further, cell-based therapeutics have also been further advanced through exploring hMSC-derived extracellular vesicle as the vehicle of choice, which may provide a means to circumvent the variability associated with hMSC cell-based delivery [32,33].

hMSCs have the potential to alter infection and inflammation through both direct and indirect interactions with the host. The production of soluble antibacterial mediators such as LL37 and other molecules such as CCL20 results in immune cell recruitment and antimicrobial activity [22,34,35,36]. Antimicrobial peptides can provide important protection in anti-inflammatory therapies in patients who have diseases characterized by concurrent infections such as CF. Our data would suggest that hMSC LL-37 is important in defining antimicrobial potency against *P. aeruginosa*; however, it is likely that the antimicrobial activity of hMSC-derived LL-37 is complimented by other hMSC-derived soluble mediators for other types of infections [13,22]. The in vivo therapeutic potency of hMSCs likely includes other AMPs and cytokine combinations that ultimately define the in vivo potency. Utilizing hMSCs as a therapeutic resource of endogenously produced human LL37 and CCL20 relies on the functional production of these antimicrobial peptides by the hMSCs. Our studies have demonstrated significant variability in LL37 (and CCL20) production, thus suggesting donor selection does provide greater potential for a therapeutic benefit in vivo [21,22]. Mechanistically, these antimicrobial peptides can alter the initial host response to infection as well as directly interact with the pathogen, making the bacteria more susceptible to antibiotics [22,34,35,36]. LL-37 is a highly complex anti-microbial peptide with the capacity to create pores into extracellular pathogens and provide a chemotactic gradient for cellular recruitment [22,37]. LL-37 may be important in providing clearance of infection and resolution of inflammation [38,39]. In follow-up studies, we will focus on monitoring soluble factors that have been directly linked to *P. aeruginosa’s* survival and activity [40,41]. hMSC secreted products, such as IDO, IL-17, IL-6, IL-8 and IL-10, re-direct host immunity by impacting macrophage and T-cell phenotypes for specific adaptive and innate mechanisms, supporting the host in the pursuit of homeostasis [42,43,44,45]. The variability in patient hMSC responses is highly complex and likely attributable to multiple factors including severity of the disease, host characteristics, and the unique phenotype of each hMSC donor preparation. Future studies are planned to evaluate unique hMSC fingerprints associated with antimicrobial and anti-inflammatory potency and to evaluate whether responses vary by disease pathophysiology.

## 4. Materials and Methods

### 4.1. Cell Sources

#### 4.1.1. hMSCs

Human posterior iliac crest bone marrow aspirates of 10–20 mL were obtained under an approved institutional review board protocol from Case Western Reserve University and University Hospitals Cleveland Medical Center (IRB, #09-90-195). Each hMSC donor preparation was screened for blood-based infectious diseases to ensure eligibility if selected as a donor for the CF clinical trial discussed in the partner paper in this journal by Roesch et al.: A Phase I Study Assessing the Safety and Tolerability of Allogeneic Mesenchymal Stem Cell Infusion in Adults with Cystic Fibrosis (J. Cystic Fibrosis, November 2022). The hMSCs were isolated and expanded in ex vivo culture as per previously published methods [46,47,48,49]. Pre-GMP procedures have been previously described, which completely mimic the GMP process without the expense of the clean room [50]. We compared standardized hMSC growth medium containing human platelet lysate to medium free of serum and platelet lysate for hMSC growth and function to optimize the growth conditions of the hMSCs. The standard platelet lysate supplemental medium was selected for the pre-clinical development of the hMSCs and the phase I clinical trial in CF [46,47,48,49]. Each hMSC donor preparation (passage 2 or 3) was grown in antibiotic-free conditions for 3 days prior to harvesting the conditioned medium (supernatant), or cells were utilized in these studies. Demographic data for de-identified hMSC preparations are presented in Table 1. HLA testing for compatibility is not required of hMSCs by the FDA because hMSCs do not express histocompatibility proteins [7].

#### 4.1.2. Human Transformed Airway Epithelial Cell Models

Airway epithelial cell lines obtained from a person with CF (ATCC: CRL-4017™, IB3) or from a healthy volunteer (ATCC: CRL-4011™, S9) and that had been validated for drug development and toxicology studies were utilized in these studies. CF airway epithelial cells secrete elevated concentrations of pro-inflammatory cytokines that are further increased when exposed to pathogen molecules such as lipopolysaccharide (LPS). Monolayers of healthy and CF epithelial cells were generated for the testing of the hMSC supernatant preparations. Primary CF patient and control airway epithelial cells grown at the air-liquid interface would provide a great modeling system for quantifying hMSC anti-inflammatory potency and are the focus of on-going studies. However, the goal of these studies is to monitor the anti-inflammatory function, which can take advantage of transformed cells since the hMSC function is expressed as the ability to change inflammation.

#### 4.1.3. Bone Marrow-Derived Macrophages (BMDM)

Bone marrow-derived macrophages (BMDM) were obtained from WT (C57BL/6j) and CF mice (*Cftr^tm1Kth^*, representing R117H on the C57BL/6j background, *Cftr^−/−^*) as previously described [21,50,51]. Briefly, hematopoietic cells from bone marrow were rinsed with 1X phosphate buffered saline and centrifuged at 1800 rpm for 9 min. After discarding the supernatant, cells were re-suspended in medium (RMPI + 10% HI-FBS + 1% PSG + 46 mls L929 conditioned medium) and plated at 2.5 × 10^6^ cells in 5ml per petri dish. The purpose of using the BMDM was to provide a link between the in vitro assays to the in vivo modeling to verify the potency testing algorithms for easy translation. Future studies could envision using peripheral blood mononuclear cells from patients and controls and quantifying hMSC impact like what was conducted for the original validation studies (13,14).

### 4.2. In Vitro Assays

#### 4.2.1. Anti-Microbial Potency Assays

The hMSCs were evaluated for anti-microbial and antibiotic potency using a clinical isolate of mucoid *Pseudomonas aeruginosa* (*P. aeruginosa*, PAM 5715) because this pathogen is highly associated with CF lung infections [21,22]. The hMSCs were grown in the presence of antibiotic free medium for 72 h prior to evaluation in antimicrobial assays. At 72 h, the medium was harvested, centrifuged to remove any cellular debris, aliquoted, and frozen at −80 °C to sustain activity. Each hMSC-CM preparation was analyzed against *P. aeruginosa* cultured the conditioned medium 1:1 in the medium used to grow the *P. aeruginosa*. Cultures were analyzed at 2 and 4 h. Anti-microbial activity was monitored by how the hMSCs altered bacteria growth [measured by colony forming units; (CFUs)]. The negative controls included the basal medium used to grow the hMSCs, and gentamicin (50 μg/mL) served as the positive control. We had previously validated the sensitivity to gentamicin control and the dynamic range of potency [5,6].

#### 4.2.2. Anti-Inflammatory Potency Assays

These potency models utilized transformed epithelial cell lines from a CF patient (IB3 and S9 cells) or bone marrow-derived macrophages from *Cftr* knockout (CF: *Cftr^tm1Kth^*) or wild type mice (WT: C57BL/6J). The inflammatory human target cell was an immortalized epithelial cell line obtained from a person with CF (*Cftr*^−/−^). Control consisted of a transformed cell transfected with an empty vector to provide paired *Cftr*^−/−^ and *Cftr*^+/+^ controls. To correlate the murine in vivo modeling with in vitro potency assays, BMDM were obtained from *Cftr*^−/−^ and *Cftr*^+/+^ mice and monitored for inflammatory response.

The target cells (epithelial or BMDM) were cultured in the presence and absence of 100 µg/mL LPS to mimic pathogen-induced inflammation as seen in CF. Both non-treated and LPS-treated cellular target inflammatory responses were quantified for each of the 8 different hMSC-conditioned media at 1:1 of cell culture medium. Cultures were harvested after 24 h with supernatants harvested, centrifuged and aliquoted. Cell was harvested and processed for RNA using RNA easy for PCR analysis. The anti-inflammatory functional potency of the hMSCs was associated with the capacity to decrease inflammatory molecule production by immune cells. The controls included non-stimulated cell targets and stimulated cell targets cultured with the medium used to grow the parent hMSCs. The inflammatory profile of the target cells (epithelial cells or macrophages) was followed for pro-inflammatory cytokine gene expression (TNFα for macrophages and IL-8 from the human transformed epithelial cells) using RT-PCR [4,5,31].

### 4.3. In Vivo Models

*Cftr*-deficient mice (*Cftr^-tm1Kth^*) were utilized to provide a modeling system that would mimic the excessive inflammatory response to *P. aeruginosa* infection as seen in CF [52,53,54,55]. These mice generally possess most of the pathophysiological sequela of CF and provide an efficient and unique platform to determine hMSC potency and in vivo efficacy. The *Cftr*^−/−^ and *Cftr*^+/+^ mice were inoculated with a validated slurry of 10^6^ CFUs of viable *P. aeruginosa* encased in agarose beads via the transtracheal route. Twenty-four hours after inoculation, hMSCs were infused at 10^6^/100 µL through the retro-orbital sinus because of their efficient clearance into the lung. Each group of infected mice (WT and *Cftr*^−/−^) were subdivided into hMSC infusion or media control. The mice were monitored daily for 10 days for changes in body weight. Mice were euthanized and evaluated for pulmonary inflammation using bronchoalveolar lavage (BAL) and quantitative bacteriology of BAL fluid and whole lung homogenate.

#### 4.3.1. LL-37 Assay

LL-37, an antimicrobial peptide is produced by hMSCs [35]. In previous studies, we demonstrated the potential relationship between the production of LL-37 by hMSCs and anti-inflammatory and anti-microbial potency using in vivo and in vitro modeling [16,21,22]. In the pursuit of the ideal donor for the “First in CF” Phase I clinical trial, hMSC LL-37 production was evaluated using an ELISA-based assay as previously described [16,21].

#### 4.3.2. Statistics

The data underwent linear or log transformation and were utilized to compare between experimental conditions using unpaired T-tests and one-way ANOVA for statistical analysis [56,57]. In the acute and chronic infection models, survival curves were compared using stratified log-rank tests with *P. aeruginosa* and hMSCs as strata. Bacterial CFUs, white cell counts, and cytokine concentrations were log-transformed as necessary to compare between groups or conditions using one or two-way ANOVA, treating donors as experimental blocks. Prism Software (GraphPad Prism 9.0.2) was utilized for analyses. One-way ANOVA and Bartlett’s correction was incorporated to account for multiple variables and correction for data normalization when required. Non-parametric ANOVA analysis was utilized to analyze the correlation between LL-37 and functional antimicrobial activity. Significance was defined as *p* ≤ 0.05.

## 5. Conclusions

In these studies, eight different pre-GMP hMSC donor preparations were interrogated for anti-inflammatory and anti-microbial potency to identify the ideal hMSC sources for the First in CF Clinical Trial of hMSCs (CEASE-CF, NCT02866721). Screening the eight pre-GMP donors using in vitro anti-inflammatory and anti-microbial assays aided in the selection of an hMSC donor with the potential to provide anti-inflammatory and anti-microbial efficacy in the clinical trial. The two most effective in vitro hMSC preparations were then evaluated in the in vivo murine model of CF lung infection and inflammation to select the potentially most efficacious donor preparation to be used in the clinical trial. The preparation of hMSC donor cells followed the requirements mandated by the FDA for human clinical trials. The systematic selection of the donor hMSC preparation for the CEASE trial was geared towards providing potency and potential efficacy against CF airway infection and inflammation. To directly correlate the in vitro and in vivo potency profiles of hMSCs to the clinical trial data in the Phase I study, future evaluations will be needed since the Phase I study only utilized one donor hMSC source.

## Figures and Tables

**Table 1:** Demographics of hMSC Donors.

Donor ID	Age	Sex
786	35	Male
822	53	Male
829	33	Male
875	37	Male
882	28	Female
TB001	36	Female
TB002	34	Male
TB003	27	Female

## Data Availability

Data are contained within the article.
